# The Macklin effect in tension pneumomediastinum in a patient with interstitial pneumonia

**DOI:** 10.1002/rcr2.1045

**Published:** 2022-09-29

**Authors:** Hiroko Watanabe, Hiroaki Ishikawa, Toshihide Inui, Kai Kawashima, Tomohiro Namiki, Tohru Sakamoto

**Affiliations:** ^1^ Department of Respiratory Medicine Tsukuba Memorial Hospital Tsukuba Japan

**Keywords:** interstitial pneumonia, Macklin effect, pneumomediastinum

## Abstract

Tension pneumomediastinum is a rare complication of interstitial pneumonia. This case shows computed tomography findings of the Macklin effect, in which air dissection along the bronchovascular interstitium caused by alveolar rupture leads to pneumomediastinum.

## Graphical abstract

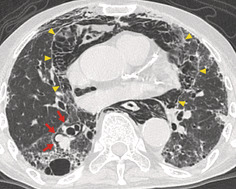

## CLINICAL IMAGE

**FIGURE 1:**
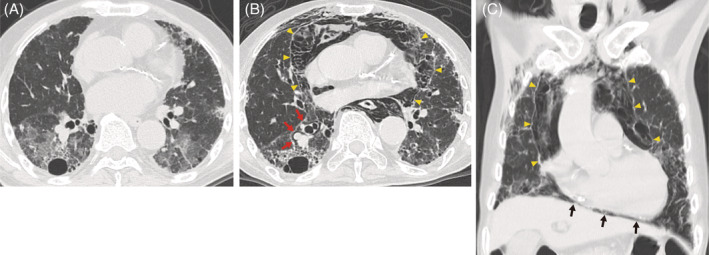
(A) Axial computed tomography (CT) at the onset of acute exacerbation of interstitial pneumonia showed bilateral ground‐glass opacities. (B) Axial CT on admission at the same level as (A) demonstrated massive pneumomediastinum (yellow arrow heads) with compression of the cardiovascular structures and air along the peribronchovascular bundle (red arrows). (C) Coronal section of the pneumomediastinum (yellow arrow heads) revealed a continuous diaphragm sign (black arrows)

An 82‐year‐old man was admitted to our hospital due to shortness of breath and chest discomfort. He did not complain of cough. During the preceding 3 months, he had been treated with steroid pulse therapy followed by a gradual tapering of oral prednisolone for acute exacerbation of idiopathic interstitial pneumonia (Figure 1A). Chest computed tomography (CT) on admission demonstrated massive pneumomediastinum (Figure 1B,C), anteroposterior compression of the heart (Figure 1B) and a continuous diaphragm sign (Figure 1C). The blood pressure was 128/88 mmHg and pulsus paradoxus was detected with a 36 mmHg fall in systolic blood pressure during inspiration. The CT scan further identified air along the peribronchovascular bundle (Figure 1B), suggestive of the Macklin effect,^1^ in which air dissection along the bronchovascular interstitium caused by alveolar rupture leads to pneumomediastinum. Pneumomediastinum with interstitial lung disease treated by corticosteroid reportedly tends to occur during the improvement of ground‐glass opacity.^2^ Our case supports this hypothesis. Tissue fragility induced by corticosteroid therapy might also have predisposed to pneumomediastinum. Since the blood pressure remained normal, he was treated conservatively and discharged after 36 days. To the best of our knowledge, this is the first report of tension pneumomediastinum with pulsus paradoxus.

## AUTHOR CONTRIBUTION

Hiroko Watanabe and Tohru Sakamoto contributed substantially to the writing of the manuscript. Hiroaki Ishikawa, Toshihide Inui, Kai Kawashima and Tomohiro Namiki contributed substantially to critical review. All authors read and approved the final manuscript.

## CONFLICT OF INTEREST

None declared.

## ETHICS STATEMENT

The authors declare that appropriate written informed consent was obtained for the publication of this manuscript and accompanying images.

## Data Availability

The data that support the findings of this study are available from the corresponding author upon reasonable request.
